# Supplementary material for: 129Xe ultra-fast Z spectroscopy enables micromolar detection of biosensors on a 1 T benchtop spectrometer
Source: Magn Reson (Gott). 2021 Jun 11;2(1):409–20. doi: 10.5194/mr-2-409-2021 (PMC10539730; doi:10.5194/mr-2-409-2021)
Supplement: The supplement related to this article is available online at: https://doi.org/10.5194/mr-2-409-2021-supplement. [file mr-2-409-supplement.pdf]

Supplement of Magn. Reson., 2, 409–420, 2021
https://doi.org/10.5194/mr-2-409-2021-supplement
© Author(s) 2021. CC BY 4.0 License.

Open Access
MAGNETIC RESONANCE

*Supplement of*

# $^{129}$Xe ultra-fast Z spectroscopy enables micromolar detection of biosensors on a 1 T benchtop spectrometer

**Kévin Chighine et al.**

*Correspondence to:* Patrick Berthault (patrick.berthault@cea.fr)

The copyright of individual parts of the supplement might differ from the article licence.

**Pulse program of the 129Xe Ultrafast Z-spectroscopy on the Magritek Spinsolve 43 C spectrometer:**

```
############################################################
#
# A pulse sequence suitable for performing an UFZ on X nuclei
# with the Spinsolve Spectrometer (P. Berthault - Jan 2021).
#
############################################################

procedure(pulse_program,dir,mode)

# Interface description (name, label, ctrl, vartype)
  interface = ["nucleus",        "Nucleus",                  "tb",  "readonly_string";
               "b1FreqX",        "X frequency (MHz)",        "tb",  "freq";
               "90AmplitudeX",   "Pulse amplitude (dB)",      "tb",  "pulseamp";
               "pulseLengthX",   "Pulse length (us)",         "tb",  "pulselength";
               "ampPs",           "Presat amplitude (dB)",     "tb",  "float,[-85,-16]";
               "pulseLengthPs",   "Presat length (ms)",        "tb",  "float,[0,1e4]";
               "b1FreqPs",        "Presat frequency (MHz)",    "tb",  "freq";
## For introducing an homospoil gradient in the sequence
#               "spoilAmp",         "Homospoil amplitude",       "tb",  "float,[0,1e4]";
#               "spoilDur",         "Homospoil duration (ms)",   "tb",  "sdelay";
               "shiftPoints",     "Number of points to shift", "tb",  "float,[-100,100]";
               "repTime",         "Repetition time (ms)",      "tb",  "reptime";
               "acquDiv",         "Acquisition",               "dv",   "";
               "rxGain",          "Receiver gain",             "tm",  "integer,[-20:3:70]";
               "rxChannel",       "Receiver channel",          "tm",
"string,[\"1H\",\"13C\",\"15N\",\"19F\",\"29Si\",\"31P\",\"X\"]";
               "rxPhase",         "Receiver phase",            "tb",  "float,[-360,360]";
               "nrPnts",          "Number of points",          "tm",
"integer,[4,8,16,32,64,128,256,512,1024,2048,4096,8192,16384,32768]";
               "dwellTime",       "Dwell time (us)",           "tm",
"float,[0.5,1,2,5,10,20,50,100,200,500,1000,2000]";
               "nrScans",         "Number of scans",           "tb",  "float,[1,1e8]";
               "gradTime",        "Gradient on time (us)",     "tbw", "sdelay";
               "gradRamp",        "Gradient ramp time (us)",   "tbw", "sdelay";
               "gradAmp",         "Read gradient amplitude",   "tbw", "float,[-165000,165000]";
               "kcat",            "Ratio G_sat/G_acq",         "tbw", "float,[-1,1]";
               "adjust",          "Adjustment delay (us)",     "tbw",  "sdelay"; #PB
               "flatFilter",      "Flat filter",               "cb",  "no,yes";
               "accumulate",      "Accumulate data",           "cb",  "no,yes";
               "usePhaseCycle",   "Phase cycle",               "cb",  "no,yes";
               "bandwidth",       "Bandwidth (kHz)",           "tb2", "float";
               "acqTime",         "Acquisition time (ms)",     "tb",  "float";

               "procDiv",         "Processing",                "dv",  "";
               "zf",              "Zero fill factor?",         "tm",  "integer,[1,2,4,8,16]";
               "filter",          "Apodisation filter?",       "cb",  "no,yes";
               "filterType",      "Filter type",               "tm",
"string,[\"none\",\"exponential\",\"sinebellsquared\"]";
               "tdPhaseCorr",     "Time. domain phasing",      "tm",
"string,[\"autophase\",\"mag\",\"none\"]";
               "fdPhaseCorr",     "Freq. domain phasing",      "tm",
"string,[\"autophase\",\"mag\",\"none\"]";
               "dispDiv",         "Display",                   "dv",  "";
               "usePPMScale",     "Use ppm scale?",            "cb",  "no,yes";
               "dispRangeMinPPM", "Minimum ppm value",         "tb",  "float,[-2000,2000]";
               "dispRangeMaxPPM", "Maximum ppm value",         "tb",  "float,[-2000,2000]";
               "dispRange",       "Display range (Hz)",        "tb",  "float,[0,2e6]";
               "filesDiv",        "Files",                     "dv",  "";
               "saveData",        "Save data?",                "cb",  "false,true"]


# Relationships to determine remaining variable values
   relationships = ["nDataPnts  = nrPnts",
                    "b1Freq     = b1FreqX",
                    "a90Amp     = 90AmplitudeX",
                    "d90Dur     = pulseLengthX",
## Homospoil gradient
#                    "nzSpoil    = zshim-sign(zshim*spoilAmp)",
#                    "nzShim     = zshim",
#                    "dSpoil     = spoilDur*1000",
                    "dRecov     = 1500",
                    "aSat       = ampPs",
                    "wSat       = 1000*pulseLengthPs",
```

```
                     "dAcqDelay  = ucsUtilities:getacqDelay(d90Dur,shiftPoints,dwellTime)",
                     "offFreq    = b1Freq",
                     "O1         = offFreq",
                     "wvPPMOffset = 0",
                     "totPnts    = nrPnts",
                     "totTime    = acqTime",
                     "n2         = 0",
                     "n3         = gradAmp",
                     "n4         = -gradAmp",
                     "n5         = n3*kcat",
                     "d300       = 2",
                     "n300       = round((gradRamp)/d300)",
                     "d3         = gradTime-gradRamp",
                     "d4         = adjust",
                     "f1         = double(b1FreqX)",
                     "f2         = 10d*f1",
                     "f3         = double(b1FreqPs)",
                     "f4         = 10d*f3"]

# These parameters will be changed between experiments
   variables = [""]

# Pulse sequence
   initpp(dir)                      # Define compile directory and clear parameter list
   settxfreq(f3)
   delay(10000)                     # Wait 10 ms allowing time to finish lock scan
   gradramp(n2,n5,n300,d300)
   txon(2,aSat,p1)
   wait(wSat)
   txoff(2)
   delay(200)
   gradramp(n5,n2,n300,d300)
   delay(50)
   pulse(2,a90Amp,p1,d90Dur)        # RF pulse on channel 2 with phase p1
   setrxfreq(f2)
#   Spoiler:Z(nzSpoil,nzShim,dSpoil,dRecov)
#   delay(50)
   gradramp(n2,n4,n300,d300)
   delay(d3)
   gradramp(n4,n2,n300,d300)
   delay(200)
   gradramp(n2,n3,n300,d300)
   delay(d4)
   delay(dAcqDelay)                 # Pulse - acquire delay
   acquire("overwrite",nDataPnts)   # Acquire FID
   gradramp(n3,n2,n300,d300)
   parList = endpp()                # Combine commands and return parameter list

# Phase cycle list
   phaseList  = [0,1,2,3;   # p1 : Pulse phase
                 0,1,2,3]   # pA : Acquire phase


endproc(parList,list(0),interface,relationships,variables,null,phaseList)
```

**Experiment control:**

```
#########################################################
#
# PBUFZ1d-129Xe
#
# A pulse sequence suitable for performing an
# UFZ on X nuclei on the Spinsolve Spectrometer.
#
# U.I. V5
#########################################################

#########################################################
#
# The is the entry point for the SpinsolveExpert
# interface. It will add the experiment to the parameter
# list or with the control key pressed open the
# relevant macros in the pulse program compiler.
#
# Autogenerated
#
#########################################################

procedure(PBUFZ1d-129Xe)

   macroLocation = getmacropath()
   parentPath = getbasepath(macroLocation)
   ppGroup = getbasedir(parentPath)

   if(iskeypressed("shift"))
      PulseProgramCompiler(guiwinnr(),null,parentPath,"PBUFZ1d-129Xe")
   elseif(iskeypressed("control"))
      gView->showExperimentHelp("PBUFZ1d-129Xe")
   else
      gExpt->addExperiment(ppGroup,"PBUFZ1d-129Xe")
   endif

endproc()

#########################################################
#
# Provide a backdoor interface to this macro. This
# adds [""] to the user interface list (guipar)
# and also generates the pulse program lists required
# by execpp. Finally is calls execpp, returning any
# results in the structure 'r'.
#
# Autogenerated
#
#########################################################

procedure(backdoor, guipar)

   seqInfo = :getseqpar()
   r = gSeq->initAndRunPP(getmacropath(), getmacroname(), guipar, seqInfo)

endproc(r)

#########################################################
#
# Returns important pulse sequence parameter lists
#
# rel ......... relationship between pulse sequence parameters
# var ......... variable which change during the pulse sequence
# pp_list ..... list of pulse sequence parameters sent to DSP
# pp_name ..... name of DSP pulse program to run
# phase_list .. phase cycling information
#
# Autogenerated
#
#########################################################

procedure(getseqpar)

   rel = ["nDataPnts  = nrPnts",
          "b1Freq     = b1FreqX",
```

```
          "a90Amp     = 90AmplitudeX",
          "d90Dur     = pulseLengthX",
          "dRecov     = 1500",
          "aSat       = ampPs",
          "wSat       = 1000*pulseLengthPs",
          "dAcqDelay  = ucsUtilities:getacqDelay(d90Dur,shiftPoints,dwellTime)",
          "offFreq    = b1Freq",
          "O1         = offFreq",
          "wvPPMOffset = 0",
          "totPnts    = nrPnts",
          "totTime    = acqTime",
          "n2         = 0",
          "n3         = gradAmp",
          "n4         = -gradAmp",
          "n5         = n3*kcat",
          "d300       = 2",
          "n300       = round((gradRamp)/d300)",
          "d3         = gradTime-gradRamp",
          "d4         = adjust",
          "f1         = double(b1FreqX)",
          "f2         = 10d*f1",
          "f3         = double(b1FreqPs)",
          "f4         = 10d*f3"]
   var = [""]
   pp_list = 
["f3","n2","n5","n300","d300","aSat","p1","wSat","a90Amp","d90Dur","f2","n4","d3","n3","d4","dAcqDelay","n
DataPnts"]
   pp_name = "PBUFZ1d-129Xe.p"
   phase_list = [0,1,2,3;0,1,2,3]

   seqInfo = struct(rel,var,pp_list,pp_name,phase_list)

endproc(seqInfo)

#########################################################
# Execute the pulse program, collecting nrScans of
# data and displaying the result in the 1D plot.
#
# This procedure can be modified to perform more
# complex functions using the passed parameters:
#
# guipar ..... all parameters from the user interface
# ppList ..... the pulse program parameter list
# pcList ..... phase-cycle list
# pcIndex .... indices of phase parameters in ppList
# varIndex ... indices of variable parameters in ppList
#
# 13-Oct-2019 CDE
#########################################################

procedure(execpp,guipar,ppList,pcList,pcIndex,varIndex)

# Make all gui parameters available
   assignlist(guipar)

# Allocate space for output data
   sumData = cmatrix(totPnts)

# Calculate suitable time and frequency axes
   tAxis = ([0:1:totPnts-1]/totPnts)*totTime*1000 # ms
   fAxis = [-totPnts*zf/2:totPnts*zf/2-1]/(totTime*zf)*1000 # Hz

# Time domain filter
   if(filter == "yes")
      flt = filters:get_filter(filterType,"FTFid",totPnts)
   else
      flt = matrix(totPnts)+1
   endif

# Get plot regions
   (prt,prf) = ucsPlot:getPlotRegions(guipar,2)
   prt->showimag("true")
   prf->showimag("false")

# Work out frequency axis scale, label and range
   (fAxisDisp,fAxisLabel,fRange) = ucsPlot:generate1DFrequencyAxis(prf, fAxis, b1FreqX, wvPPMOffset, 
offFreq, guipar)
```

```
# Initialise progress bar
   :updateProgress(-1,guipar)

# Accumulate scans
   for(scan = 0 to nrScans-1)

      t1 = time()

     # Set phases for this scan
       (ppList,pAcq) = ucsRun:setPPPhase(ppList,scan,pcList,pcIndex)

     # Send all parameter values to DSP
        ucsRun:updatePPParameters(ppList,guipar,wvPort)

     # Run the pulse program and collect the data
        ucsUtilities:suspendLock() # turn lock control loop off
       (status,data) = ucsRun:getData(totPnts,guipar)
        ucsUtilities:resumeLock()  # turn lock control loop on

     # See if stop button/escape key pressed
       if(status != "ok")
          return(0)
       endif

     # Shift the data to minimise p1
       data = shift(data,round(shiftPoints))

     # Correct the distortions in start of FID due to digital filter
       data = ucsUtilities:correctFilter1(data,dwellTime)

     # Accumlate the data
       sumData = ucsRun:accumulate(accumulate,pAcq,sumData,data)

     # Correct the first data point
       datacorr = sumData
       if (shiftPoints == 1)
          datacorr = ucsUtilities:correctFirstPointAmpPhase(sumData)
          datacorr[0] = datacorr[0]/2
       endif

    # FID autophase
       phCor = phase(datacorr[0])
       datacorr = datacorr*exp(-i*phCor)

     # Process data
       (phasedTimeData,spectrum,ph0) =
ucsRun:transformData(zerofill(datacorr.*flt,zf*totPnts,"end"),fAxis,guipar,"fid")

     # Simple offset baseline correction
    #   spectrum = ucsRun:baselineCorrection(spectrum,"offset",size(spectrum)/32)

     # Plot the data
       ucsPlot:graphTimeAndFreq(prt,prf,tAxis,datacorr,fAxisDisp,spectrum,scan,guipar,
                                "Time data (scan : $scan+1$)","Spectral data",
                                "Time (ms)","Amplitude (\G(m)V)",
                                fAxisLabel,"Amplitude")

     # Update progress bar
       :updateProgress(scan,guipar)

    # Check timing
       check = ucsRun:checkTimeAndAbort(guipar,t1,scan,pcList,"ignoreLastScan")
       if(check == "abort")
          return(0)
       elseif(check == "finish")
          scan = scan+1
          exitfor()
       endif

   next(scan)

# Save the data
   ucsFiles:savePlot(prt,:getPlotInfo("pt1"),guipar,"noReport")
   ucsFiles:savePlot(prf,:getPlotInfo("pt2"),guipar,"simpleReport")
   ucsFiles:saveMNovaData(prt,"",guipar,"simpleReport")
```

```
# Save the processing parameters
   :saveProcPar(guipar,ph0,fRange)

# Pack the data into a structure
   result = struct()
   result->tAxis = tAxis
   result->tData = sumData/scan
   result->fAxis = fAxisDisp
   result->fData = spectrum/scan
   result->par = struct(guipar)

# Return result
   return(result)

endproc("execpp") # Don't remove argument

#####################################################
# Assign those parameters which should take their
# values from the factory defaults when making a
# new experiment
#####################################################

procedure(getFactoryBasedParameters, par)

   specPar = SpinsolveParameterUpdater:readDSPPar(null)
   if(specPar == null)
      return(null)
   endif
   assignlist(specPar)

   modelPar = ucsUtilities:getModelBasedParameters("X",specPar)

   par = ["rxGain = $modelPar->rxGain$",
          "pulseLengthX = $Pulse_length_X$",
          "90AmplitudeX = $Power_level_X$",
          "b1FreqX = $Frequency_X$"]

endproc(par)

########################################################
# Get the name of a plot file given the region name
# or return the whole list
########################################################

procedure(getPlotInfo,plotRegion)

   info = ["pt1","fid.pt1","pt2","spectrum.pt1"]

   if(plotRegion == "all")
      return(info)
   endif

   idx = getlistindex(info,plotRegion)
   if(idx != -1)
      return(info[idx+1])
   endif

endproc(null)


########################################################
# Update the progress bar and experiment times
########################################################

procedure(updateProgress, scans, guipar)

# Define progress/timing expressions

   if(isvar("progressCtrl"))

      if(isvar("wvUpdateProgressCtrl"))
         if(wvUpdateProgressCtrl == 0)
            return
         endif
      endif

      assignlist(guipar)
```

```
   # Define progress/timing expressions
      totTime = nrScans*repTime/1000
      expTime = (scans+1)*repTime/1000
      remTime = totTime - expTime
      progress = 100*expTime/totTime

   # Update controls
     ucsCtrl:updateProgress(scans+1,progress,totTime,expTime,remTime)

   endif

endproc()

#################################
# Save the processing parameters
#################################

procedure(saveProcPar,guipar,p0,xrange)

   assignlist(guipar)

   if(saveData == "false")
      return
   endif

   if(usePPMScale == "yes")
      xrange = xrange*single(b1FreqX)
   endif

   procpar = ["apodizationFunction = \"$filterType$\"",
              "baseLineCorrectionMethod = \"None\"",
              "displayInPPM = \"$usePPMScale$\"",
              "ftOrigin = \"Start\"",
              "ftType = \"Complex\"",
              "p0Phase = $p0$",
              "p1Phase = 0",
              "p1Pivot = 0",
              "p1FixedPhase = 0",
              "phaseMethod = \"p0, p1 fixed phase\"",
              "ppmOffset = $centerFreqPPM$",
              "zeroFill = $zf$",
              "plotWidth = $xrange[1]-xrange[0]$",
              "plotStart = $xrange[0]$",
              "shiftPoints = 1"]

   cd("$dataDirectory$\\$expName$")

   if(isfile("proc.par"))
      par = load("proc.par")
      procpar = mergelists(procpar,par)
   endif

   save("proc.par",procpar)

   if(isfile("proc_temp.par"))
      rmfile("proc_temp.par")
   endif

endproc()
```

**Default parameters:**

```
90AmplitudeX = 0
accumulate = "yes"
acqTime = 102.4
adjust = 50
ampPs = -50
b1FreqPs = 12.0908299999999990d
b1FreqX = 12.0925469999999960d
bandwidth = 10
centerFreqPPM = 0
dataDirectory = ""
dispRange = 500
dispRangeMaxPPM = 400
dispRangeMinPPM = -400
duration = 17.4336
duration = 3.86377
duration = 34.3631
duration = 8.43994
dwellTime = 100
experiment = "PBUFZ1d-129Xe"
expName = ""
fdPhaseCorr = "mag"
filter = "yes"
filterType = "exponential"
flatFilter = "yes"
gradAmp = -20000
gradRamp = 200
gradTime = 2000
incExpNr = "no"
kcat = -0.5
nrPnts = 1024
nrScans = 1
nucleus = "X"
offFreq = 0
offFreqPs = 0
percentageCompleted = 100
percentageCompleted = 100
percentageCompleted = 100
percentageCompleted = 100
pulseLengthPs = 2500
pulseLengthX = 150
repTime = 2800
rxChannel = "X"
rxGain = 61
rxPhase = 0
saveData = "true"
shiftPoints = 1
softwareVersion = "1.40.9"
specID = ""
specType = ""
spoilAmp = 7000
spoilDur = 5
tdPhaseCorr = "none"
usePhaseCycle = "yes"
usePPMScale = "yes"
zf = 1
```
